# Atrial High-Rate Episodes and Subclinical Atrial Fibrillation: State of the Art and Clinical Questions with Complex Solutions

**DOI:** 10.31083/j.rcm2508305

**Published:** 2024-08-22

**Authors:** Carola Griffith Brookles, Roberto De Ponti, Vincenzo Russo, Matteo Ziacchi, Gemma Pelargonio, Michela Casella, Maurelio Lauretti, Manola Vilotta, Sakis Themistoclakis, Antonio D’Onofrio, Giuseppe Boriani, Matteo Anselmino, on behalf of AIAC

**Affiliations:** ^1^Cardiology Division, Department of Medical Sciences, “Città della Salute e della Scienza” Hospital, Univerisity of Turin, 10116 Turin, Italy; ^2^Department of Medicine and Surgery, University of Insubria, 21100 Varese, Italy; ^3^Cardiology Unit, Department of Translational Medical Sciences, Monaldi Hospital, University of Campania “Luigi Vanvitelli”, 80131 Naples, Italy; ^4^Institute of Cardiology, IRCCS Azienda Ospedaliero Universitaria di Bologna, 40138 Bologna, Italy; ^5^Fondazione Policlinico Universitario Agostino Gemelli IRCCS, University “Cattolica del Sacro Cuore”, 00168 Rome, Italy; ^6^Cardiology and Arrhythmology Clinic, Department of Clinical, Special and Dental Sciences, Marche Polytechnic University, Marche University Hospital, 60126 Ancona, Italy; ^7^Cardiology Division, “Vito Fazzi” Hospital, 73100 Lecce, Italy; ^8^Department of Heart and Vessels, Ospedale di Circolo, 21100 Varese, Italy; ^9^Department of Cardiothoracic, Vascular Medicine and Intensive Care, Dell'Angelo Hospital, 30174 Mestre-Venice, Italy; ^10^Electrophysiology and Cardiac Pacing Unit, Cardiology Division, Monaldi Hospital, 80131 Naples, Italy; ^11^Cardiology Division, Department of Biomedical, Metabolic and Neural Sciences, University of Modena and Reggio Emilia, Policlinico di Modena, 41124 Modena, Italy

**Keywords:** atrial high-rate episode, subclinical atrial fibrillation, cardiac implantable electronic devices, thromboembolic risk, cognitive impairment, anticoagulation

## Abstract

Atrial high-rate episodes (AHREs) and subclinical atrial fibrillation (AF) are frequently registered in asymptomatic patients with cardiac implantable electronic devices (CIEDs) and insertable cardiac monitors (ICMs). While an increased risk of thromboembolic events (e.g., stroke) and benefits from anticoagulation have been widely assessed in the setting of clinical AF, concerns persist about optimal clinical management of subclinical AF/AHREs. As a matter of fact, an optimal threshold of subclinical episodes’ duration to predict stroke risk is still lacking and recently published randomized clinical trials assessing the impact of anticoagulation on thromboembolic events in this specific setting have shown contrasting results. The aim of this review is to summarize current evidence regarding classification and clinical impact of subclinical AF/AHREs and to discuss the latest evidence regarding the potential benefit of anticoagulation in this setting, highlighting which clinical questions are still unanswered.

## 1. Introduction 

Atrial fibrillation (AF) is the most common sustained cardiac arrythmia in the adult population [1, 2]. The lifetime risk of AF in European individuals is estimated as 1 in 3, with an increasing incidence starting from the age of 50 years in males and 60 years in females, reaching a cumulative incidence of roughly 30% by the age of 90 years. Considering progressive ageing of the population, AF is estimated to affect more than 17.9 million people in Europe by 2030. The link between AF and increased incidence of thromboembolic events, namely transient ischemic attack (TIA), overt ischemic stroke, peripheral embolism and silent embolic lesions, has been assessed [3]. Independently from these events, AF is associated with a 30% increased risk of cognitive decline and dementia [4, 5], whose pathophysiology still needs to be fully clarified.

Considering its clinical impact and increasing prevalence, AF has become a prominent public health issue, prompting the need for a rapid diagnosis and correct clinical management. While diagnosis is straightforward in symptomatic patients, identification of asymptomatic patients is often achieved during rhythm monitoring after cerebrovascular accidents (CVAs) [6], questioning the need for AF screening, or occasionally at surface electrocardiogram (ECG). Furthermore, the increasing number of patients with cardiac implantable electronic devices (CIEDs) capable of atrial rhythm monitoring, as well as insertable cardiac monitors (ICMs), has led to frequent detection of atrial high-rate episodes (AHREs) or subclinical AF [7]. When these episodes are not associated with surface ECG documentation, there is uncertainty about correct clinical management and anticoagulation.

In the present review, we discuss the epidemiological and clinical impact of atrial fibrillation, providing insights into the latest research regarding the pathophysiological link between AF, cognitive decline and dementia. Subsequently, we focus on subclinical AF and AHREs with regard to their definition and impact on thromboembolic risk. Eventually, we summarize the latest evidence concerning use of anticoagulants in this specific setting, highlighting clinical issues that persist unsolved.

## 2. Definitions

Despite being frequently encountered in clinical practice, confusion in terminology is still widespread when it comes to classifying AF. According to the latest guidelines [8], the definition of clinical AF implies the recording of a 12-lead surface ECG or at least 30 second single-lead tracing, documenting irregular R-R intervals and the absence of P waves. Depending on symptoms, clinical AF can be distinguished by being symptomatic or asymptomatic. The real proportion of asymptomatic patients is difficult to assess, varying from 10 to 40% between studies, depending on patients’ features, duration of follow-up and modality of screening [9, 10, 11]. However, asymptomatic AF is more frequent in male patients and when arrhythmia is persistent [9, 12].

On the other hand, the definition of AHRE and subclinical AF has been extremely heterogeneous in literature, both in terms of atrial rate and episode duration cut-offs, starting from any atrial tachyarrhythmia with an atrial rate >180 beats per minute (bpm) lasting for at least 5–6 minutes [13], to any atrial event with an atrial rate >190 bpm independent of duration [14].

According to the European Society of Cardiology (ESC) guidelines [8], the definition of AHRE implies the presence of an atrial tachyarrhythmia with an atrial rate >175 bpm lasting for at least 5 minutes, detected by CIEDs with an atrial lead or atrial sensing capacity, in patients who are asymptomatic and who do not have a history of AF. When stored electrograms are reviewed to exclude artifacts, double counting, or noise they can be referred to as subclinical AF. Despite this distinction, the two terms are frequently used interchangeably. Definition of AHRE implies the inclusion of different kinds of atrial tachyarrhythmias, with a variable degree of organization and cycle length (such as focal atrial tachycardia, supraventricular reentry tachycardia, AF and typical/atypical atrial flutter), without excluding the shift from one to the other.

According to guidelines [8] it is the single-episode duration to be considered in AHRE definition. However, it is important to introduce the concept of “AHRE/subclinical AF burden”, which is defined as the overall time spent in AHRE during a certain period, usually 24 hours. Guidelines suggest that both elements should be considered when trying to predict thromboembolic risk, as the dynamic entity of AHRE implies a progressive increase in episodes’ duration and daily or monthly burden during follow-up, as well as progression to clinical AF [15]. Despite the lack of a specific single episode duration to predict a thromboembolic event, the latest guidelines suggest that a single episode duration of 24 hours should be the cut off to consider use of oral anticoagulants (OACs), particularly in the presence of a high monthly burden [8].

## 3. Neurocognitive Impact of Atrial Fibrillation: Beyond Overt Thromboembolic Events.

Cardioembolic ischemic stroke is the most dreaded complication of AF. It can be AF’s first clinical manifestation in otherwise asymptomatic patients, while AF is detected in 25–30% of patients with embolic stroke of unknown source (ESUS), rising questions on the need for AF screening [16]. Despite diffusion of effective acute treatments, stroke is still associated with a dramatic increase in the risk of developing dementia, and cognitive impairments are found in nearly 70% of stroke survivors [17].

In the latest years, it has been demonstrated that AF is associated with a significant increase (30%) in the risk of developing cognitive decline/dementia independently from stroke or TIA, even in patients receiving OACs [5]. Despite overlapping risk factors, the relationship between AF and cognitive impairment persists after adjustment for these variables [18]. However, controlled trials are essential to prove the existence of a causal relationship between AF and cognitive impairment. The presence of causality seems to be favored by a possible biological gradient between arrhythmic burden and dementia, as suggested by the Rotterdam study [19], in which there was a correlation between quantitative exposure to AF and new-onset dementia in young patients. These results are consistent with those of the ARIC study, in which persistent, rather than paroxysmal, AF was associated with lower cognitive performances assessed through validated scores [20]. However, it remains to be established what the minimal amount of arrhythmic burden is, which is associated with cognitive decline and whether OAC therapy is efficient in preventing it independently from stroke [21]. Interestingly, the two trials designed to assess the potential benefit of OAC therapy on stroke risk in patients with AHREs deal with this issue. The ARTESIA trial [22] incorporates a cognitive substudy in which patients are periodically evaluated through cognitive assessment scales, and cognitive function changes are included in the secondary outcomes of the NOAH-AFNET 6 trial [23].

Several mechanisms have been proposed to explain the link between AF and cognitive decline in the absence of CVAs. First, silent cerebral lesions (SCLs) due to micro-embolic events are found in a high proportion of patients with AF and a negative anamnesis for stroke or TIA undergoing cerebral imaging, such as magnetic resonance. When clinically silent lesions are represented by large non-cortical and cortical infarction, they correlate with cognitive decline [24]. Another potential mechanism is represented by microbleeds [25], which are more frequently encountered in patients with AF compared to those in sinus rhythm. Their presence has been linked to an increased risk of mortality, intracranial hemorrhage and stroke [26].

More recently, researchers have been concentrating on the hemodynamic consequences of AF [27], especially focusing on cerebral perfusion. Using phase contrast MRI, Gardarsdottir *at al*. [28] demonstrated a reduction of cerebral blood flow and estimated brain tissue perfusion in patients with AF. Interestingly, cerebral blood flow reduction was reversible after 10 weeks from effective cardioversion, with a documented increase in tissue perfusion assessed through both MRI and arterial spin labeling [29]. Similarly, a small prospective study demonstrated a significant increase in cerebral blood flow assessed with phase-contrast MRI after successful ablation with maintenance of sinus rhythm beyond a blanking period of three months [30].

Based on data from two closed lump models simulating AF and sinus rhythm, Saglietto *et al*. [31] proposed that beat-to beat variability during AF results in the alternation of micro-hypertensive and micro-hypoperfusion events in distal cerebral circulation. It has been hypothesized that the observed high-variability in hemodynamic parameters could result in microbleeds and infarctions, and therefore in the development of cognitive decline. Impact of beat-to-beat variability in cerebral perfusion has been validated *in vivo* using spatially resolved near-infrared spectroscopy (SR-NIRS) [31], which showed that both hypoperfusive and hyperperfusive events at the microcirculatory level were reduced after restoration of sinus rhythm through electrical cardioversion (*p* < 0.001 and *p* = 0.041), without significant changes in arterial blood pressure.

When SCLs are found, they are usually located at the subcortical level and involve the white matter. Considering lenticulostriate arteries (LSAs) are the main blood supply of this area, it was hypothesized that these terminal vessels could be particularly exposed to extreme hemodynamic events determined by AF’s “irregularly irregular” rhythm. Computational studies evaluating the effects of irregular AF rhythm, compared to sinus rhythm, on wall shear stress and intraluminal pressure along these vessels [32]. Results showed that the irregular AF rhythm exposes LSAs to both an increased range of wall shear stress, and to a wider range of intraluminal pressure along their course. The excessive oscillations from shear stress in both directions have been associated with both a pro-atherogenic effect [33] and acute complications, such as plaque erosion and rupture [34]. Similarly, oscillations in intraluminal pressure during hypertensive states can lead to brain barrier damage and accelerate lypohyalinosis, resulting in lacunar stroke; on the other hand, reduction of intraluminal pressure can provoke hypoperfusion and subsequent ischemia [35]. Coherently with these observations, a cognitive benefit from rhythm control would be expected. The AFFIRM [36] and EAST AFNET 4 [37] trials compared rate and rhythm control strategies in patients with AF. Neither AFFIRM (in which rate control was pursued through drug therapy) nor EAST AFNET 4 (in which catheter ablation was allowed) showed a benefit in cognitive functions in the rhythm control group. However, in the AFFIRM trial only 63% of patients in the rhythm control group were in sinus rhythm at 5-year follow-up [38]. Furthermore, more recent studies and metanalyses have shown an advantage of rhythm over rate control on dementia outcome (subdistribution hazard ratio (sHR) 0.86, 95% confidence interval (CI) 0.80–0.93 and hazard ratio (HR) 0.60, 95% CI 0.42–0.88 respectively) [39, 40]. Overall, evidence is still conflicting and relies on retrospective studies with possible selection bias. Furthermore, SCLs are a potential complication of catheter ablation itself, mostly represented by small, cortical lesions [41]. Randomized clinical trials (RCTs) are necessary to clear contrasting evidence; however, they are difficult to organize, considering difficulties in early cognitive decline assessment and the need for long-term follow-up.

## 4. Epidemiology and Clinical Implications of AHRE

It is difficult to define true prevalence of AHREs in patients carrying a CIED. Despite definitions introduced by guidelines throughout the years, criteria to identify AHRE remain heterogeneous between studies. Furthermore, the prevalence of AHREs not only depends on the chosen cut offs in terms of atrial rate and duration, but also on population features, indication for implantation of devices, arrhythmia recognition algorithms and duration of follow-up (Table 1, Ref. [6, 42, 43, 44, 45, 46, 47, 48, 49, 50, 51, 52, 53, 54, 55, 56]). Overall, AHREs are quite common in the CIED population, and episodes lasting more than 5 minutes are found in a proportion of patients varying between 10 and 68% [7, 42]. When considering only studies excluding patients with previous clinical AF, overall prevalence of AHRE is lower (approximately 30%) [43, 57, 58, 59].

**Table 1.** **Definitions and prevalence of AHREs and subclinical AF across different studies**.

Author and year	Number of patients	Type of device	AHRE/Subclinical AF definition	Exclusion if clinical AF documented	Mean follow-up	Patients with AHRE/Subclinical AF
Caldwell *et al*. 2009 [44]	162	CRT	Any mode-switch occurrence on the device with an atrial rate ≥200 bpm lasting for at least 30 seconds.	NA	14.1 months	16.6%
Bertini *et al*. 2010 [45]	393	ICD, CRT	Any AT with an atrial rate >180 lasting for at least 10 minutes in patients with CRT/Dual-chamber ICD. In single- chamber devices, with device-based diagnostics.	No	16 months	21.3%
Petrač *et al*. 2012 [46]	308	Dual chamber PM	Any AT with an atrial rate >220 bpm lasting for at least 5 minutes.	Yes	36 months	24.6%
Healey *et al*. 2012 [ASSERT] [42]	2580	Dual chamber PM, CRT, ICD	Any AT with an atrial rate >190 bpm lasting for more than 6 minutes (required EGM confirmation).	Yes (if lasting more than 5 minutes)	30 months	10.1%
Witt *et al*. 2015 [43]	394	CRT	Any AT (according to manufacturer-specific nominal settings) lasting for at least than 6 minutes.	Yes	50 months	20.0%
Martin *et al*. 2015 [47]	2718	ICD, CRT-D	Any AT with an atrial rate ≥200 bpm lasting for at least 6 minutes.	Yes (permanent)	23 months	21.0%
Kim *et al*. 2016 [48]	880	PM, ICD and CRT	Any AT with an atrial rate ≥180 beats/min lasting for at least 5 minutes (in dual chamber CIED). Device-based diagnostic for single chamber CIED.	Yes	52.2 months	13.8%
Van Gelder *et al*. 2017 [ASSERT] [49]	2455	Dual chamber PM, ICD	Any AT with an atrial rate ≥190/min lasting for at least 6 minutes (required EGM confirmation).	Yes	30 months	36.3%
Amara *et al*. 2017 [SETAM] [50]	595	PM	Any AT with an atrial rate ≥190/min lasting for at least 6 minutes (required EGM confirmation).	Yes	12.8 months	25%
Kawakami *et al*. 2017 [51]	343	Dual chamber PM	Any AT with an atrial rate >175 bpm lasting for at least 6 minutes.	Yes (permanent and persistent)	52 months	48.1%
Kaplan *et al*. 2019 [52]	21,768	Dual chamber PM, ICD and CRT	AT/AF lasting for at least 6 minutes.	No	NA	22.7%
Li *et al*. 2019 [53]	594	PM, ICD, CRT	Any AT with an atrial rate ≥175 bpm lasting for at least 5 minutes.	Yes	50.4 months	29.4%
Miyazawa *et al*. 2019 [54]	856	Dual chamber PM, ICD, CRT	Any AT with an atrial rate ≥175 lasting for at least 5 minutes (required EGM confirmation).	No	48.2 months	14.6%
Nakano *et al*. 2019 [55]	348	Dual chamber PM, ICD, CRT	Any AT with an atrial rate >175, 190, and 200 beats/min according to the Medtronic, Abbott, and Biotronik devices, respectively, lasting for at least 30 s (required EGM confirmation).	Yes	65 months	21.5%
Nishinarita *et al*. 2019 [56]	104	Dual chamber PM	Any AT with an atrial rate >170 bpm lasting for more than 5 minutes (required EGM confirmation).	Yes	65 months	32.6%
Lu *et al*. 2021 [6]	355	PM	Any AT with an atrial rate >175 bpm (Medtronic) or >200 bpm (Biotronik) and lasting for at least 30 seconds (required EGM confirmation).	Yes	42.1 months	45.6%

AF, atrial fibrillation; AHREs, atrial high-rate episodes; AT, atrial tachyarrhythmia; CIED, cardiac implantable electronic device; CRT-P/D, cardiac resynchronization therapy-pacemaker/defibrillator; EGM, electrogram; ICD, implantable cardioverter defibrillator; PM, pacemaker; NA, not available.

AHREs and subclinical AF represent a dynamic entity. In the Asymptomatic Atrial Fibrillation and Stroke Evaluation in Pacemaker Patients and the Atrial Fibrillation Reduction Atrial Pacing Trial (ASSERT) [42], which identified AHREs as any atrial tachyarrhythmia (atrial rate >190 bpm) lasting at least 6 minutes, prevalence of subclinical events increased from 10% to 25% from 3-month to 2.5-year follow-up, while 16% of patients with AHREs developed clinical symptomatic AF. Not only the prevalence in CIED population rises extending the follow-up, but also AHRE burden tends to increase over time, as well as the duration of single episodes. In a pooled metanalysis of TRENDS (A Prospective Study of the Clinical Significance of Atrial Arrhythmias Detected by Implanted Device Diagnostics), PANORAMA and SOS AF projects [15], approximately 40% of patients with subclinical AF progressed to a higher daily burden of AHRE at 6-month follow up; the greater the burden of subclinical AF at first detection was, the faster transition to a higher burden happened. A CHA2DS score >2 and male sex were independently associated with a faster transition to AHRE burden >23 hours. Furthermore, it has been highlighted how AHREs can trigger chronic atrial changes [60], configuring the so-called atrial cardiomyopathy [61], an umbrella term which includes atrial abnormalities such as atrial fibrosis, endothelial damage, atrial enlargement and impaired contractility, all related to an increased risk of stroke independent from AF. Interestingly, not only AF can trigger atrial remodeling, but such atrial abnormalities increase the risk of developing atrial tachyarrhythmias, which could be interpreted as markers of a pro-thrombotic atrial substrate [62].

Patients with AHREs are at higher risk of developing clinical AF. In a metanalysis considering 2892 patients from ASSERT and Ancillary Mode Selection Trial (MOST AHREs), AHREs were associated with a 5.7 times (95% CI 4.0–8.0, *p* < 0.001) increase in likelihood of documenting clinical AF during follow-up. However, 38% of patients from Ancillary MOST [63] had previous supraventricular tachycardias.

## 5. Implication of Subclinical AF/AHREs on Thromboembolic Risk

Ischemic stroke, which can be the first clinical manifestation of AF, plays a detrimental contribution to its morbidity and mortality. It is estimated that nearly 30% of ischemic strokes are related to AF; this is the reason why long-term cardiac rhythm monitoring should be implemented to detect arrhythmia in patients with cryptogenetic stroke [64]. Furthermore, cardioembolic strokes are usually multifocal and involve large cerebral territories, resulting in significant neurologic sequelae [65].

AF-related stroke risk is not mitigated in asymptomatic patients. When comparing symptomatic and asymptomatic patients (12%) in the Atrial Fibrillation Follow-up Investigation of Rhythm Management (AFFIRM) study, no significant difference was found in terms of mortality and major events after adjusting for baseline features [66]. Similarly, in a sub-study of the Prevention of Thromboembolic Events—European Registry (PREFER) in AF Registry there was no difference in the incidence of ischemic stroke or TIA between symptomatic and asymptomatic patients. Despite these observations, prescription of anticoagulants is lower in this particular subset of patients [9].

Considering the well-assessed link between stroke and clinical AF, which is independent from symptoms, one of the main interests regarding AHRE/subclinical AF was assessing its possible relationship with increased thromboembolic risk and the subsequent need for anticoagulation.

In the Registry of Atrial Tachycardia and Atrial Fibrillation Episodes (RATE Registry) [43], very short AHREs (defined as AHRE in which the onset and offset of the arrhythmic event were within the same electrogram (EGM), lasting between 15 and 20 seconds) did not correlate with an increase in adverse clinical events, including stroke, at a follow up of nearly 2 years. However, despite lack of uniformity in AHRE definition, numerous studies considering longer episodes have highlighted that patients with AHREs display an increased thromboembolic risk, whose entity varies across study groups [42, 45, 47, 49, 50, 51, 52, 54, 55, 63, 67, 68, 69, 70, 71, 72, 73, 74, 75, 76].

When considering data from more than 10,000 patients’ using the Italian Clinical Service Project, PANORAMA and TRENDS [76], AHRE burden resulted as an independent predictor of stroke after adjusting for the CHA2DS2VASc score. After testing different cut offs, a 1-hour AHRE burden was associated with a significant increase in the risk of ischemic stroke (2.11; 95% CI 1.22–3.64, *p* = 0.008). However, absolute stroke risk in the AHRE population was low (0.39% annual rate). A metanalysis [77] considering various cut offs in terms of AHRE rate, duration, and burden, has shown that patients with subclinical AF lasting more than the study-specific cut off had a 2.4-fold increase (95% CI 1.8–3.3, *p* < 0.001, I^2^ = 0%) in stroke risk when compared to patients with AHREs shorter than the cut-off duration (between 5 minutes and 24 hours depending on studies) or without AHRE. Annual stroke rate in patients with AHRE single episodes or burden lasting more than pre-specified cut-off duration was 1.89/100 person-year (95% CI 1.02–3.52).

In a recent metanalysis including more than 61,000 patients with CIEDs and insertable loop recorders (ILRs), AHREs lasting more than 30 seconds as well as day-level cumulative duration lasting more than 24 hours were associated with a significant increase in the risk of stroke and systemic embolism (HR, 4.41; 95% CI 2.32–8.39); the increase in stroke risk persisted across longer single episodes’ cut off duration (5-minutes, 6-hours and 24-hours thresholds), while no association was found between episodes shorter than 30 seconds and thromboembolic events [70]. Furthermore, both linear and non-linear meta-regression did not suggest an increase in the risk of stroke or systemic embolism considering progressively longer AHRE thresholds. Overall, stroke risk in patients with subclinical AF was lower than clinical AF, especially when considering historical cohorts [78].

In the assessment of AHRE-related thromboembolic risk, clinical information should be taken into consideration as well. In a study by Botto *et al*. [75], patients were stratified not only on the basis of subclinical AF daily burden (<5 minutes, between 5 minutes and 24 hours, >24 hours), but also according to thromboembolic risk (assessed through the CHADS2 score). At a medium follow-up of 1 year, patients with an AHRE >5 min and CHADS2 <2, or AHREs >24 h and CHADS2 <1 had a higher annual risk of thromboembolic events than patients with AHREs <5 min and CHADS2 <2, AHREs <24 h and CHADS2 <1, or AHREs >24 h and CHADS2 = 0 (5% vs. 0.8%; *p* = 0.035).

A recently published multiple cut-off diagnostic metanalysis [79] aiming at defining an optimal threshold for AHRE duration to predict thromboembolic events, identified an extremely short duration threshold when considering both single episode duration and daily burden (0.07 minutes and 1.4 minutes per day, respectively). Furthermore, it confirmed that thromboembolic events are uncommon in the CIED population (3.0%, 95% CI 2.2–4.0). Finally, studies have failed to demonstrate a clear temporal association between AHREs and thromboembolic events. Considering data from the ASSERT trial [49], which enrolled 2,580 patients with CIED and no history of AF, only 8% of patients registered AHREs in the 30 days preceding stroke or other thromboembolic events; AHRE was only present in 1 patient at the time of the event. In a sub-group analysis of TRENDS [80] considering 40 patients with CIEDs, half of patients with stroke had experienced at least an episode of AHRE before the clinical event, but nearly 45% of them did not have any episodes in the 30 days before the clinical event. Therefore, it is unsolved whether AHRE should be considered in a binary or continuous manner. Altogether, these data suggest that AHRE could be interpreted as a marker of stroke risk, rather than its direct cause, and that relationship between AHREs and thromboembolic risk could be independent from episodes’ duration. Furthermore, besides assessing the link between AHREs and thromboembolic events, it would be necessary to deal with the potential neurocognitive impact of AHREs and its long-term implications.

## 6. Thromboembolic Events, AHREs and Atrial Cardiomyopathy

Interestingly, AHREs show a complex yet strict connection with atrial cardiomyopathy (ACM). ACM has been defined as “any complex of structural, architectural, contractile or electrophysiological changes affecting the atria with the potential to produce clinically relevant manifestations” [61]. ACM refers to a mixture of structural, functional, and electrical alterations in the atria which can be triggered by cardiovascular risk factors, as well as cardiac and extracardiac comorbidities (heart failure, neuromuscular disorders) [81]. In recent years, increasing interest in this clinical entity derived from evidence that alterations in atrial contractility and progressive fibrosis could result in an increase in the thromboembolic risk independently from the presence of AF [82]. Establishing the independent contribution of ACM to stroke and other embolic manifestations is complex, considering the mutual relationship between AHREs/AF and ACM. AHRE may be a signal of progressive atrial electrical derangement, however, the correlation between AHRE burden and ACM extension must be further investigated [70]. Even though it has been proven that patients with ACM have a higher thromboembolic risk independent from the presence of AHRE/AF, the Atrial Cardiopathy and Antithrombotic Drugs in Prevention After Cryptogenic Stroke (ARCADIA) trial [83] enrolling patients with a history of cryptogenic stroke, ACM (defined on the basis of ECG P wave abnormalities, echocardiographic left atrium enlargement and elevated NT-proBNP levels) and no history of AF at the time of enrolment, was prematurely interrupted due to futility for benefit of OAC therapy (apixaban 5 mg or 2.5 mg) vs aspirin in stroke recurrence (HR, 1.00 [95% CI 0.64–1.55]). Currently, ACM does not represent an indication to start anticoagulation [8].

## 7. Clinical Management of Thromboembolic Risk in Patients with AHRE: Current Indications from Guidelines

Despite being frequently encountered in clinical practice [7], management of AHREs is still a matter of debate, especially when addressing the potential need for anticoagulation. Even though it has been established that AHREs are associated with an increase in thromboembolic risk, incidence of systemic embolism is not comparable to that of clinical AF [77]. Furthermore, a definite threshold of a single episode duration or burden to distinguish between innocent bystanders and episodes with a significant impact on stroke risk has not been established [79].

When deciding whether to start an OAC or not, clinical AF is considered in a binary way (presence or absence of arrhythmia). Independent from the arrhythmic pattern (paroxysmal, persistent, or permanent AF), the start of anticoagulants (preferably direct anticoagulant oral agents, DOACs) relies on the thromboembolic risk profile, assessed through validated risk scores (mainly CHA2DS2VASc score), without distinguishing between symptomatic and asymptomatic patients [7]. An annual absolute risk for stroke >2% is identified as the cut off to recommend the start of anticoagulation therapy, which should be considered in patients with intermediate annual absolute risk (1–2%) as well [64].

Despite the absence of a clear linear relationship between AHREs/subclinical AF and stroke risk, the approach suggested by the latest guidelines support consideration of these events in a continuous manner. AHRE duration, both in terms of single episodes and daily burden, coupled with the individual risk profile defined through CHA2DS2VASc score, should be considered when deciding whether to start anticoagulants or not. According to both ESC [8] and AHA [64] (American Heart Association) guidelines, starting anticoagulation therapy requires shared decision-making, taking into consideration AHRE duration, monthly burden and ischemic risk profile. In a subanalysis of the ASSERT trial [49], adjudicated AHREs lasting more than 24 hours correlated with a significant increase in the risk of thromboembolic events, including ischemic stroke (adjusted HR, 3.24 [95% CI 1.51–6.95]; *p* = 0.003). Based on these observations, guidelines state that anticoagulation therapy may be considered in patients with a high risk of stroke (CHA2DS2VASc ≥2 in men and ≥3 in women) who have long AHREs (> 24 hours) and a high monthly burden, especially when episodes have been adjudicated by a clinician.

When the duration of AHREs is limited (<5 minutes) and patients’ individual risk for stroke is low, the start of anticoagulation therapy is typically withheld. However, considering the dynamic pattern of AHREs, it is essential to observe for an increase in duration of single episodes and burden, as well as for progression to clinical AF. In this field, remote monitoring is a useful tool to ensure a strict monitoring of burden in high-risk patients and reduce time to action in case of need [84]. Likewise, periodic re-evaluation of patient’s stroke risk is essential to detect any change that could suggest an early start of anticoagulation.

Absence of clear indications and cut offs on if and when to start anticoagulation therapy in patients with CIEDs and detection of AHREs results in great heterogeneity in clinical practice. Perception of thromboembolic risk related to AHREs is variable, and prescription of OACs depends on the clinical scenario, with high prescription rates in patients with previous stroke [85]. Furthermore, balancing between thrombotic and hemorrhagic risk is pivotal when considering anticoagulation in fragile patients who are at increased risk of bleeding complications [86, 87]. In a cohort study [73] including data of patients with CIEDs from the Veterans Health Administration coupled with remote monitoring information about daily subclinical AF burden, there was great heterogeneity in OAC prescription after subclinical AF detection. Overall, treatment rates were low (30%), even when considering patients with long episodes (>24 hours).

## 8. Randomized Clinical Trials on AHREs and Anticoagulation: NOAH-AFNET 6 and ARTESIA

**Fig. 1.**
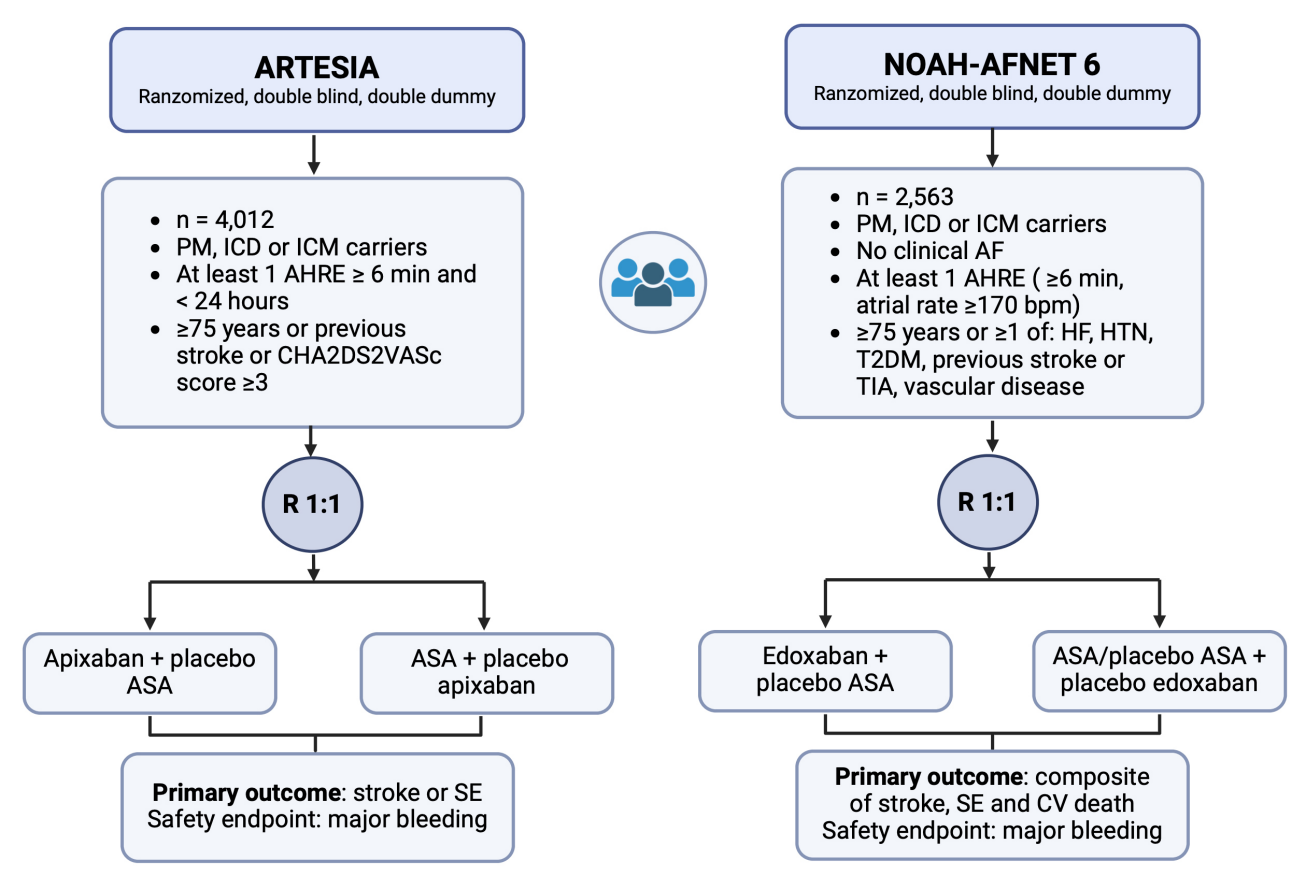
**A visual comparison between RCTs: ARTESIA and NOAH AFNET 6**. Details of the two RCTs assessing the potential benefit of OAC (apixaban and edoxaban, respectively) versus aspirin or placebo on stroke prevention in patients with at least one episode of AHRE lasting more than 6 minutes. AHRE, atrial high-rate episodes; ASA, aspirin; CV, cardiovascular; HF, heart failure; HTN, hypertension; ICD, implantable cardioverter defibrillator; ICM, insertable cardiac monitor; OACs, oral anticoagulants; PM, pacemaker; SE, systemic embolism; T2DM, type 2 diabetes mellitus; TIA, transient ischemic attack; AF, atrial fibrillation; RCTs, randomized clinical trials.

Recommendations of the latest guidelines on the use of OACs in patients with AHRE have been formulated awaiting for results of two large RCTs specifically addressing this issue: the NOAH AFNET 6 [23] (Non–Vitamin K Antagonist Oral Anticoagulants in Patients with Atrial High Rate Episodes) trial and the ARTESIA [88] (Apixaban for the Reduction of Thrombo-Embolism in Patients with Device-Detected Subclinical Atrial Fibrillation) trial (Fig. 1).

NOAH AFNET 6 is an event-driven, double-blind, double-dummy RCT which enrolled 2536 patients with CIEDs and ICMs, aged 65 years or older who had at least a risk factor for stroke on top of AHREs with a duration >6 minutes. Patients were randomly assigned to receive either edoxaban (60 mg or 30 mg daily according to guidelines) or placebo vs aspirin in those who had an indication for single antiplatelet therapy. The primary outcome was a composite of cardiovascular (CV) death, stroke and systemic embolism, while the safety outcome was a composite of all-cause death and major bleeding according to International Society on Thrombosis and Haemostasis (ISTH) criteria. Mean duration of AHREs was 2.8 hours, while patients had a median CHA2DS2VASc score of 4. The trial was interrupted prematurely due to futility for benefit of OAC therapy on the primary outcome (hazard ratio, 0.81; 95% CI 0.60 to 1.08; *p* = 0.15) and concerns about increased bleeding risk in patients receiving edoxaban (hazard ratio, 1.31; 95% CI 1.02 to 1.67; *p* = 0.03). It has to be highlighted that, despite high-risk features of study population, the incidence of stroke was lower than expected in both groups [88, 89], a phenomenon which was interpreted as a consequence of short duration and burden of AHRE episodes. Nonetheless, detection of further benefit from edoxaban could have been limited by insufficient power of trial.

ARTESIA is a double-blind, double-dummy RCT which enrolled 4012 patients with CIEDs and ICMs with episodes of subclinical AF lasting from 6 minutes to 24 hours. Any patient displaying AHREs longer than 24 hours or developing clinical AF was excluded from analysis and started on open label OACs. Patients were randomly assessed to receive either apixaban 5 mg twice daily (or 2.5 mg when indicated according to guidelines) or aspirin (81 mg daily). The primary efficacy outcome was the incidence of stroke or systemic embolism, while the primary safety outcome was major bleeding, defined according to ISTH criteria. Compared to a general population of patients with subclinical AF, those enrolled in the trial were less likely to have experienced a previous stroke or TIA. Mean CHA2DS2VASc score was 3.9 ± 1.1. The intention-to-treat analysis on primary outcome revealed a significant reduction in stroke and systemic embolism in patients aimed at receiving apixaban compared to aspirin (hazard ratio, 0.63; 95% CI, 0.45 to 0.88; *p* = 0.007). Differences between the two groups were similar when considering ischemic stroke and stroke from any cause; furthermore, strokes were classified as disabling or fatal (according to the Modified Rankin Scale, score 3–6) in 33% of patients in the apixaban group and 43% of patients in the aspirin group (HR, 0.51; 95% CI 0.29 to 0.88). Conversely, apixaban resulted in a 1.8 increase in major bleedings in the on-treatment analysis (HR, 1.80; 95% CI 1.26 to 2.57; *p* = 0.001), without a significant increase in fatal bleeding or intracranial hemorrhages. In most cases, bleeding events required treatment with conservative measures or transfusion support, while only 10% of bleeding events required immediate procedural measures. Nonetheless, the use of aspirin in the control group could have blunted the effect of OACs on safety, itself increasing bleeding risk. Collectively, in patients aimed at receiving aspirin, the risk of stroke or systemic embolism was 1.24% per patient-year, thus significantly lower than what expected for clinical AF [66]. However, in nearly half of patients with AHREs not receiving OACs neurologic sequelae were permanent.

After being long awaited, results of RCTs on the use of OACs in patients with AHREs, despite appearing as contrasting, suggest a solution to complex clinical questions which have been long unanswered. Due to early interruption, NOAH-AFNET 6 failed to demonstrate a benefit in the primary outcomes for patients with AHREs receiving edoxaban. However, the inclusion as part of the primary outcome of cardiovascular death, which strongly depends on patients’ comorbidities and underlying cardiac diseases and is only partly related to stroke, together with the selection of a population with lower thromboembolic risk compared to the ARTESIA trial, could have decreased the chance of detecting the benefit of anticoagulants [90]. Conversely, in the ARTESIA trial, patients receiving apixaban experienced a reduction in the risk of stroke and systemic embolism, at the cost of an increase in bleeding events. Even though such a result in safety outcomes was predictable, it remained to be established whether patients with AHRE have a net clinical benefit from receiving DOACs. Investigators highlighted how AHRE-related strokes, despite infrequent, were associated with permanent disability and neurologic sequalae in a significant proportion of patients (nearly 50%). On the contrary, bleeding events, even though more common in patients receiving both OACs, were frequently manageable with conservative measures and transfusions, without threatening survival or requiring urgent invasive procedures.

A recently published metanalysis [91] demonstrated that results of the two RCTs are consistent, showing a significant reduction in ischemic stroke in patients on OAC (RR 0.68, 95% CI 0.5–0.92, I^2^ = 0%), at the cost of an increase in major bleeding. No impact on cardiovascular death and all-cause mortality was found. However, patients with long AHREs (lasting at least 24 hours) were underrepresented in RCTs, as they were excluded from ARTESIA and represent a minority in NOAH-AFNET 6.

Taking all these aspects into consideration, the two RCTs offer evidence-based information for individualized decision-making for the use of DOACs in patients with AHRE. Anticoagulants have proved to be effective in reducing the risk of stroke, which is frequently disabling and fatal also in patients with subclinical AF, at the cost of an increased number of bleeding events, which were managed conservatively in more than 90% of cases [90]. Considering that stroke is perceived as a worse outcome than death in analyses evaluating patients’ preferences and perception [92], we suggest that a very careful, individually-based decision-making process, with patients’ education on the risk-benefit ratio of anticoagulation, as well as consideration of individual preferences, is essential when deciding whether to start an anticoagulation therapy in patients with AHREs. We believe that patients with device-detected subclinical AF should be conscious that OACs can consistently reduce the risk of disabling or fatal stroke, with limited and manageable adverse bleeding events. However, the decision to start OAC therapy must not obscure the need for comorbidities and bleeding risk factor assessment and modification. Considering that a significant proportion of patients with device-detected subclinical AF progress to clinical AF, especially when single episodes last more than 24 hours [23], and that progression results in an increased risk of stroke, periodical surveillance, and re-assessment of arrhythmic burden, with closer follow-ups or remote monitoring, is essential. In summary, considering NOAH AFNET 6 trial’s underpower to detect a benefit of OACs on stroke incidence, evidence suggests that OACs can be beneficial in patients with AHREs who have additional risk factors for stroke.

**Fig. 2.**
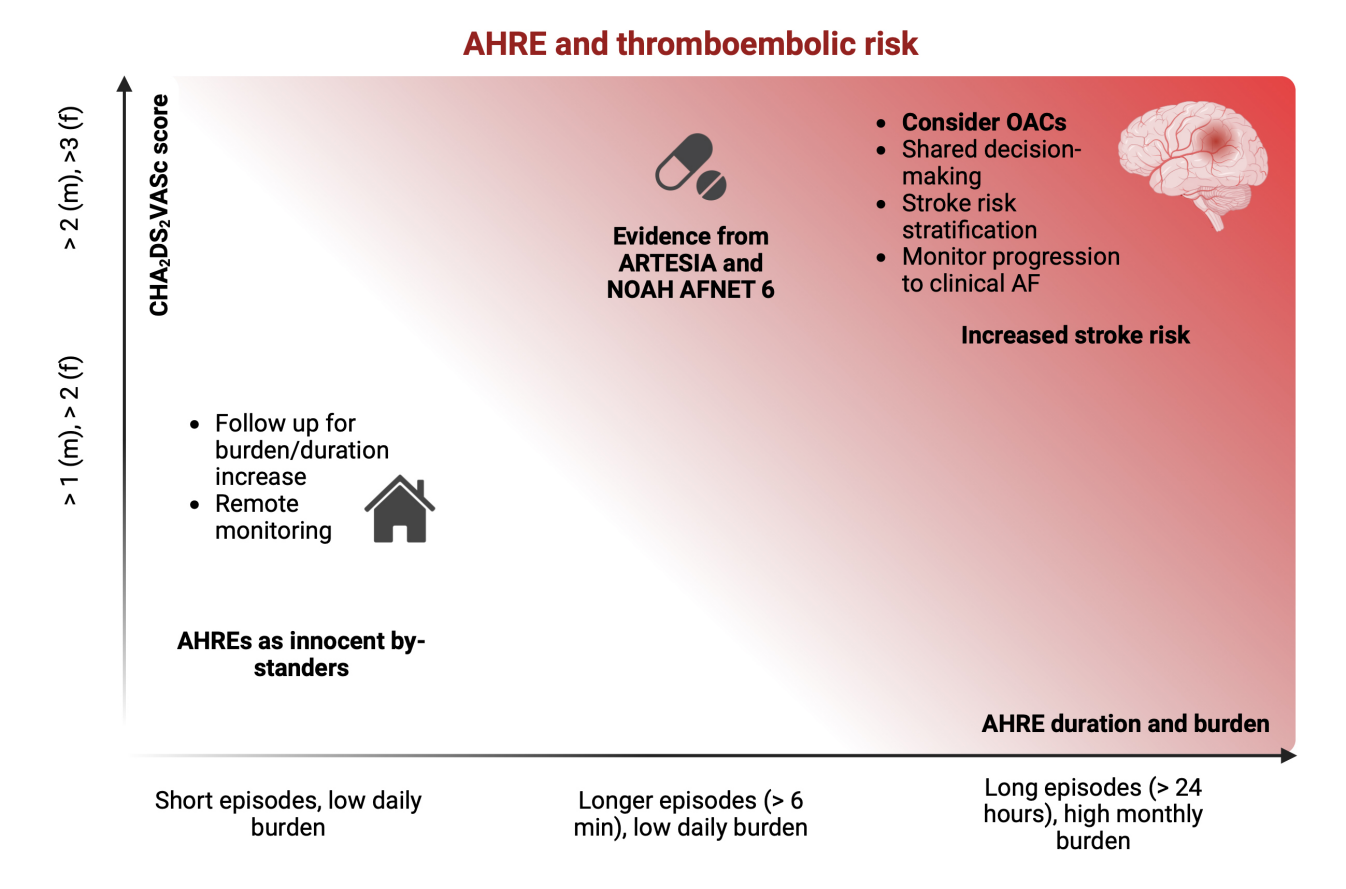
**Relationship between AHRE and thromboembolic risk**. Even though a cut off duration for AHRE to predict thromboembolic events has not been identified, studies have shown that patients with either longer single-episode duration (especially when >24 hours) or higher cumulative burden are at increased risk of stroke. Latest guidelines suggest to weight in both these aspects and consider the start of anticoagulation therapy in patients with episodes >24 hours and a high monthly burden, after balancing between embolic and hemorrhagic risk. When single episodes are short and the daily burden is low, close follow-up is necessary to detect progression to a higher burden or clinical AF, possibly with the use of remote monitoring. Latest guidelines were released before the results of the two RCTs assessing the potential benefit of OACs in patients with episodes >6 minutes (and shorter than 24 hours in the ARTESIA trial). As discussed in the review, results from trials show a significant benefit of OACs in terms of reduction of stroke risk, particularly fatal or disabling stroke. This reduction is counterbalanced by an increase in major bleeding events, which could be managed conservatively in nearly all cases. AF, atrial fibrillation; AHRE, atrial high-rate episode; f, feminine; m, masculine; OACs, oral anticoagulants; RCTs, randomized clinical trials.

As previously discussed, AHREs seem to be a marker of risk rather than a direct cause of stroke [80], and a duration threshold to predict thromboembolic events has not been identified [79]. The ARTESIA and NOAH AFNET 6 trials enrolled patients with AHREs lasting more than 6 minutes, without assessing an eventual correlation between AHREs’ duration and benefits from receiving OACs. Therefore, we are waiting for subgroup analysis to define whether a cut-off of AHRE duration or any other characterization at baseline could help identify patients who are likely to experience a greater clinical benefit from the start of anticoagulation therapy (Fig. 2).

## 9. Conclusions

Atrial fibrillation is deemed to become a major public health issue in the coming years, and its well-established relationship with cognitive decline urges an effort to unveil underlying mechanisms and limit its incidence. Despite recent evidence regarding the impact of the irregular AF rhythm on cerebral hemodynamics as a potential mechanism of silent cerebral lesions, prevention of overt stroke and systemic embolism still represents the backbone of clinical management. Notwithstanding the increase in thromboembolic risk in patients with subclinical AF, overall incidence of stroke is lower than in patients with clinical AF. Studies have failed to identify a unanimous cut off for subclinical arrhythmic events to predict thromboembolic risk, while demonstration of a temporal relationship with stroke is lacking. Overall, evidence suggests that AHREs could represent a marker of risk rather than a direct cause of stroke. Recently published randomized clinical trials exploring the effect of OACs on prevention of thromboembolic events in patients with AHREs (lasting more than 6 minutes) have shown a benefit from the use of OACs on stroke risk, despite an expected increase in major but not fatal bleeding events, which could be managed conservatively in more than 90% of cases. Albeit infrequent, AHRE-related strokes are frequently associated with permanent neurologic sequalae, suggesting a net clinical benefit could derive from use of OACs despite an increase in bleeding events. Further studies are needed to clear these aspects and to establish whether a cut-off of AHRE duration could help identify patients who may benefit from OACs.
